# Respiratory Morbidity in Children with Repaired Congenital Esophageal Atresia with or without Tracheoesophageal Fistula

**DOI:** 10.3390/ijerph14101136

**Published:** 2017-09-27

**Authors:** Maria Francesca Patria, Stefano Ghislanzoni, Francesco Macchini, Mara Lelii, Alessandro Mori, Ernesto Leva, Nicola Principi, Susanna Esposito

**Affiliations:** 1 Paediatric Highly Intensive Care Unit, Department of Pathophysiology and Transplantation, Università degli Studi di Milano, Fondazione IRCCS Ca’ Granda Ospedale Maggiore Policlinico, 20122 Milan, Italy; francesca.patria@policlinico.mi.it (M.F.P.); stefano.ghislanzoni@studenti.unimi.it (S.G.); mara.lelii87@gmail.com (M.L.); alessandro.mori@studenti.unimi.it (A.M.); nicola.principi@unimi.it (N.P.); 2 Unit of Paediatric Surgery, Fondazione IRCCS Ca’ Granda, Ospedale Maggiore Policlinico, 20122 Milan, Italy; francesco.macchini@gmail.com (F.M.); ernesto.leva@policlinico.mi.it (E.L.); 3 Paediatric Clinic, Department of Surgical and Biomedical Sciences, Università degli Studi di Perugia, 06123 Perugia, Italy

**Keywords:** congenital esophageal atresia, pediatric pulmonology, respiratory morbidity, tracheoesophageal fistula

## Abstract

Congenital esophageal atresia with or without tracheoesophageal fistula (CEA ± TEF) is a relatively common malformation that occurs in 1 of 2500–4500 live births. Despite the refinement of surgical techniques, a considerable proportion of children experience short- and long-term respiratory complications, which can significantly affect their health through adulthood. This review focuses on the underlying mechanisms and clinical presentation of respiratory morbidity in children with repaired CEA ± TEF. The reasons for the short-term pulmonary impairments are multifactorial and related to the surgical complications, such as anastomotic leaks, stenosis, and recurrence of fistula. Long-term respiratory morbidity is grouped into four categories according to the body section or function mainly involved: upper respiratory tract, lower respiratory tract, gastrointestinal tract, and aspiration and dysphagia. The reasons for the persistence of respiratory morbidity to adulthood are not univocal. The malformation itself, the acquired damage after the surgical repair, various co-morbidities, and the recurrence of lower respiratory tract infections at an early age can contribute to pulmonary impairment. Nevertheless, other conditions, including smoking habits and, in particular, atopy can play a role in the recurrence of infections. In conclusion, our manuscript shows that most children born with CEA ± TEF survive into adulthood, but many comorbidities, mainly esophageal and respiratory issues, may persist. The pulmonary impairment involves many underlying mechanisms, which begin in the first years of life. Therefore, early detection and management of pulmonary morbidity may be important to prevent impairment in pulmonary function and serious long-term complications. To obtain a successful outcome, it is fundamental to ensure a standardized follow-up that must continue until adulthood.

## 1. Introduction

Congenital esophageal atresia with or without tracheoesophageal fistula (CEA ± TEF) is a relatively common malformation that occurs in 1 in 2500–4500 live births, with males having a slightly increased incidence [1,2,3,4]. It is mostly a sporadic event with low familial recurrence. Moreover, in approximately 50% of cases, CEA ± TEF is associated with additional anatomic anomalies [5]. Heart, genitourinary, gastrointestinal, musculoskeletal, and central nervous system defects are encountered in 35%, 24%, 24%, 13%, and 10% of cases, respectively [6]. Most infants have more than one malformation. In 36% of cases, these may occur as part of the VACTERL spectrum (vertebral anomalies, anorectal atresia, congenital heart malformations, tracheoesophageal fistula, renal abnormalities, and limb defects) or CHARGE syndrome (coloboma, heart defects, atresia choanae, retardation of growth and/or development, genital hypoplasia, and ear deformities). Trisomies of chromosomes 18 and 21, Di George syndrome and Pierre Robin syndrome are other significant risk factors for CEA ± TEF [7,8,9]. These underlying diseases may be detected before birth.

Usually, symptoms of CEA ± TEF appear in the first hours of life because the neonate is unable to swallow saliva and needs repeated suctioning to aspirate oral secretions. However, oral feeding is not possible because it causes emesis, cough, and respiratory distress. Treatment must be implemented as soon as possible and consists of early surgical correction of CEA by primary anastomosis of the esophageal stumps and closure of the TEF, if it is present, as well as by specific therapies for management of the complications. The thoracotomy repair remains the most common approach [10], although in recent years, approximately 10% of all cases have been corrected by minimally invasive surgery via thoracoscopy [11], which seems to offer advantages in terms of low rates of rib fusion and subsequent scoliosis [12]. The improvement of surgical techniques in children with CEA has been associated with an overall increase in survival rates, which now exceeds 90% [13]. However, despite the refinement of surgical techniques, a considerable proportion of children experience short- and long-term respiratory complications, which can significantly affect their health through adulthood [14]. Generally, short-term pulmonary sequelae occur in the early postoperative period, whereas long-term morbidity is mostly associated with the original malformation itself and its consequences on other organs. This review focuses on the underlying mechanisms and clinical presentation of respiratory morbidity in children with repaired CEA ± TEF.

## 2. Early Respiratory Morbidity

The reasons for the short-term pulmonary impairments are multifactorial and related to the surgical complications, such as anastomotic leaks, stricture, and recurrence of fistula [15]. Anastomotic leakage generally occurs in 10–20% of the cases [16,17] and can result from the weakness of the segment related to an excess of anastomotic tension or recurrent infections [18]. Leakage is one of the most important risk factors for life-threatening complications, including serious systemic infections, severe pneumonia, atelectasis, pleural effusion, and tension pneumothorax [19,20]. In a study by Zhao et al., conservative management (i.e., chest-tube drainage, suspension of feedings, total parenteral nutritional support, and administration of broad-spectrum antibiotics) was applied, and anastomotic leaks developed in 21/85 neonates (25%) with CEA [21]. The authors isolated mainly highly resistant Gram-negative bacteria (i.e., *Escherichia coli*, *Klebsiella pneumoniae*, *Acinetobacter baumannii*, *Pseudomonas aeruginosa*, *Enterobacter cloacae*) from the pleural drainage fluid of 12/21 newborns. To prevent anastomotic leaks, some authors have suggested the early use of broad-spectrum antibiotics and continuous suctioning of the upper pouch, parenteral nutrition, or trans-anastomotic tube feeding and postoperative elective ventilatory support [22,23].

The anastomotic stricture is another common esophageal complication (30–50% of cases), which can be a risk factor for short-term respiratory impairment. It is characterized by an intrinsic circumferential narrowing of the esophageal lumen, which is related to the presence of gastro-esophageal reflux (GER), tension of the anastomosis, and long-gap atresia [24]. The common clinical presentations of anastomotic stricture include dysphagia, regurgitation, aspiration pneumonia, and chronic respiratory tract infections [25]. The best treatment remains esophageal dilatation, although some children may require more than one maneuver because the strictures usually recur [26]. Endoscopic steroid injection, systemic steroid administration, or the topical application of mitomycin C are considered adjuvant therapeutic strategies in addition to the dilation of the recurrent or refractory strictures [27].

Recurrent TEF is a frequent (5–10%) complication of CEA ± TEF repair [28]. It generally occurs in the early postoperative period but can sometimes remain undiagnosed for many years. It is more frequent in the case of anastomotic leakage, but infection may also play a role in the recurrence [29]. Manifestations of recurrent TEF include chronic cough, choking, cyanosis with feeding, dyspnoea, wheezing, and recurrent pneumonia [30]. In the case of clinical suspicion, tracheobronchoscopy and esophagography are mandatory. Spontaneous closure is rare, and therefore, this complication requires a new surgical intervention [31]. Table 1 summarizes the reasons for the short-term pulmonary impairments and their clinical manifestations.

## 3. Long-Term Respiratory Morbidity

With the improvement of surgical and post-surgical care, most children with CEA ± TEF reach adulthood. However, a substantial amount of cases suffers from respiratory problems including bronchiectasis, with a frequency significantly higher than those without CEA ± TEF [28]. These findings can persist into adulthood, require gastric tube dependence and are likely to be multifactorial, depending either on the original malformation itself or on the consequences of the malformation in other organs. A study conducted in 101 adult patients who had undergone surgical repair of CEA with or without TEF showed that after 22–56 years of follow-up they had more respiratory symptoms and infection, as well as asthma and allergies, and more often poorer respiratory symptom-related quality of life than age-matched controls [32]. Generally, long-term respiratory morbidity is grouped in four categories according to the body section or function primarily involved: upper respiratory tract, lower respiratory tract, gastrointestinal tract, and aspiration and dysphagia (Table 2).

## 4. Upper Respiratory Tract Involvement

### 4.1. Tracheomalacia

Tracheomalacia is a common finding in children who have undergone surgery for CEA ± TEF, with a prevalence ranging from 37.5% to 75% of patients [33,34], and the more severe expression of the disease is reported in 11–33% of children [35]. The impaired cartilage integrity usually corresponds to the TEF entrance, but it can sometimes be more extensive [36]. Tracheomalacia is secondary to congenital defect in tracheal cartilage development and tracheal weakness results in dynamic tracheal collapse, especially during the expiratory phase when the intrathoracic pressure physiologically exceeds the intraluminal tracheal pressure [37].

The functional impairment depends on the length of the involved segment and the degree of airway collapse. The typical symptoms are brassy cough and expiratory stridor. In most cases, they are self-limiting, with spontaneous resolution in the first months/years of life [38]. However, in the most serious cases, tracheomalacia can interfere with ventilation, leading to severe respiratory obstruction, especially during crying, when very high intrathoracic pressure is generated. Additionally, during feeding the dilation of the esophagus may worsen the tracheal collapse. Moreover, severe tracheomalacia can significantly impair the clearance of respiratory secretions, resulting in a non-efficacious cough and ineffective elimination of secretions [39]. In severe cases, affected children present with feeding difficulties, recurrent pulmonary infections with respiratory failure, and severe episodes of upper respiratory tract obstruction until life-threatening crises of apnea occur—the so-called ‘apneic spells’. ‘Apneic spells’, also known as cyanotic spells, blue spells, dying spells, or apparent life-threatening events, refer to a bluish tone visible in the mucosal membranes and skin caused by an oxygen decrease in the peripheral circulation [40]. Although this decrease may be transient and benign, it may also be indicative of a severe underlying problem that requires immediate intervention. Significant airflow reduction can be successfully treated by maintaining the patency of the upper airways through non-invasive positive pressure ventilation [41]. In severe cases, the surgical intervention of aortopexy is recommended [42]. More rarely, patients with very severe tracheomalacia unresponsive to surgical correction may require long-term tracheostomy with a long cannula to support tracheomalacia as the children grow older [43].

### 4.2. Vocal Cord Dysfunction

In children with aero-digestive malformations, vocal cord paresis or paralysis, although uncommon, can occur after surgical repair [44,45,46,47,48]. Bilateral paralysis is a potentially life-threatening condition because it can lead to complete airway obstruction, requiring long-term tracheostomy placement, whereas the mildest cases may resolve spontaneously [44,45,46,47,48]. Moreover, vocal cord paralysis can also be lateralized and not lead to airway obstruction [44,45,46,47,48]. However, all these children are at increased risk of swallowing dysfunction and aspiration [47,48]. Their ability to swallow has to be systematically evaluated to reduce risks of respiratory complications.

The incidence of vocal cord dysfunction in patients who have undergone surgical repair varies significantly among studies and is reported in 3% to 30% of the cases according to the criteria used to evaluate the problem [44,45,46]. A recent Italian retrospective analysis documented that postoperative vocal cord paralysis is more common in patients with type B CEA (CEA, with a proximal TEF, or a TEF connecting between the upper pouch of the esophagus and the trachea) and E CEA (TEF connecting between the esophagus and the trachea, without CEA) [47]. Moreover, the thoracoscopy repair seemed to be associated with a higher rate of vocal cord impairment [48]. However, it is likely that not all these cases are caused by the surgical repair due to prolonged intubation or recurrent laryngeal or vagus nerve injury during surgery. Many cases can be the result of a congenital malformation [44]. Differentiation between congenital and acquired cases is difficult because evaluation of vocal cord function via laryngoscopy is not routinely performed pre-operatively, although some authors suggest that the procedure be performed before CEA ± TEF repair [49].

## 5. Lower Respiratory Tract Involvement

### Chest Wall Deformities

The standard open technique of postero-lateral thoracotomy is commonly employed in infants for repair of CEA ± TEF [50]. This surgical approach, requiring rarely muscle and rib resection, is associated with many long-term issues with an impact to the chest wall, such as scoliosis, shoulder girdle muscle weakness and chest wall deformity. In particular, severe scoliosis has been reported in a percentage ranging from 6% to 50% of cases, generally five or more years after the thoracotomy [51,52]. The chest wall deformity has an adverse effect on lung growth and may cause restrictive lung disease with long-term development of chronic respiratory insufficiency.

The more recent thoracoscopic approach using minimally invasive techniques has significantly reduced the extent of the chest wall complications [53]. However, vertebral anomalies may also be pre-existing, as a part of syndromic pattern. In a large study of adult patients, vertebral anomalies were detected in 45% of the cases, most commonly in the cervical spine (38% of the patients). The most significant risk factor for vertebral anomalies was any additional anomaly [5].

## 6. Gastrointestinal Involvement

### Esophagus Dysmotility and Gastro-Esophageal Reflux Disease (GERD)

Gastro-esophageal reflux disease (GERD) is one of the most frequent complications encountered in patients with repaired CEA ± TEF, with a reported prevalence ranging from 22% to 55% [54,55]. The primary symptoms are recurrent vomiting, heartburn, regurgitation, growth failure in children and an apparent life-threatening event in infants. Moreover, although no study has evaluated the real correlation between GERD after CEA ± TEF repair and pulmonary symptoms, GERD is associated, especially in children, with pulmonary complications, including cough (8–75%), wheezing (14–40%), bronchitis (14–74%), recurrent pneumonia (5–50%), atelectasis (up to 90%), bronchiectasis (17%), and airway hyperreactivity (33–65%) [55,56].

Many structural and functional factors are probably involved in the pathogenesis of GERD after surgery, although a congenital and permanent impairment in the motor function of the esophagus seems to be the most relevant condition. In a recent review, Tovar and Fragoso underlined the abnormality of extrinsic and intrinsic esophageal innervations, leading to defective motor function and sphincters [56]. However, post-surgical modifications of the gastro-esophageal junction, such as shortened intra-abdominal segment and reduction of the His angle, may play a role in GERD development [57]. Symptoms of GERD can appear immediately after surgical repair or become evident some months later but may be a lifelong problem. Koivusalo et al. studied 61 patients who underwent repair for CEA with endoscopy and pH-metry and found GERD in 16.3%, 39.3%, 44.2%, 51.2%, and 44.4% of the patients at 6 months, 1 year, 3 years, 5 years, and 10 years, respectively [58]. In adults with CEA ± TEF, respiratory problems become less frequent, whereas gastro-intestinal symptoms remain very common [59]. In particular, symptoms of dysphagia and heartburn are reported in a high percentage of cases, with a frequency of 39–85% and 34–63%, respectively [60,61,62]. There is also evidence that persistent GERD with chronic acid exposition is associated with permanent scarring and damage to the esophagus, which can lead to columnar metaplasia and increase the risk of esophageal cancer [63]. In two studies, this has been described with a prevalence of 0.8% and 2.6%, respectively. Moreover, biopsy proven Barrett’s esophagus is reported in five studies with a prevalence ranging from 1.1% to 11.3% with a pooled estimated prevalence obtained from a meta-analysis of various studies of 6.4%. For these reasons, long-term periodic monitoring is advisable also in adulthood [64]. The gold standard tests for GERD diagnosis are 24-h period pH-test and pH-impedance test, in which first test monitors the esophageal acid exposition and the second test quantifies and correlates the non-acid reflux with symptoms. In a recent study conducted on 22 children treated for CEA, it was shown that in children younger than one year, the majority of clinically-relevant refluxes were not of acid [65]. In another recent retrospective study, approximately 50% of children had GERD detected only with multichannel intraluminal impedance-pH monitoring, which has been underestimated if only conventional pH monitoring has been used [66]. In select cases, when pH monitoring shows normal results and GERD symptoms persist, a barium contrast study and endoscopy are also required. In addition to general non-surgical management of GERD including sleeping position and type of food, gastric acid suppression with proton pump inhibitors is the first-line treatment for GERD, but the drug of choice, the dosage, the optimal length, the success rate and the risks of this therapy are not well determined. Moreover, no drugs are effective in the treatment of non-acid reflux and surgical fundoplication becomes necessary up to 45% of patients, particularly in cases with refractory anastomotic stricture and in those with pure and long-gap CEA ± TEF [61]. Nevertheless, this procedure can worsen distal esophageal motility and can lead to a worsening of respiratory complications [60].

## 7. Aspiration and Dysphagia

### Swallowing and Feeding Problems

Dysphagia is a common symptom related to CEA, with an incidence, in children and adolescents who underwent surgical repair, ranging between 21% and 84% [61,63,67]. Like GERD, dysphagia recognizes a multi-factorial etiology, including congenital esophageal dysmotility, GERD, and esophagitis. Additionally, surgical intervention can damage the esophagus smooth muscle and innervation and may lead to recurrent stenosis and esophageal diverticula, which, in turn, can contribute to dysphagia [61]. This condition is often associated with swallowing dysfunction, heartburn, regurgitation, and early sensation of postprandial fullness.

Many children with dysphagia need to eat slowly, mostly avoiding solid foods, and need to drink a substantial amount of water to help swallowing [67,68]. In some cases, feeding refusal can influence nutritional status and growth development. In these severe cases esophageal manometry can be used. In a recent retrospective evaluation of 75 children with repaired CEA, malnutrition and stunting were reported in 9% of cases, respectively, especially in the first years of life [69]. There is also a relationship between dysphagia and the development of recurrent lower respiratory tract infections because dysphagia can cause tracheal aspiration. Aspiration is an underrecognized cause of feeding difficulty in children with EA [28,59]. Using a feeding questionnaire, Puntis et al. showed the presence of coughing or choking during feeds in 20% (25/124) of children born with CEA ± TEF, and Smith et al. reported the presence of food sticking in the esophagus in 18/23 (78%) of children who underwent repair [70,71,72].

If dysphagia is suspected, an endoscopic evaluation of the esophagus should be performed to identify anatomical anomalies, such as strictures, diverticula, or esophagitis signs. Esophageal manometry is also recommended for identifying esophageal dysmotility, especially in those patients with normal endoscopic features [56]. To assess the presence of aspiration during swallowing, the videofluoroscopic swallow study is helpful in differentiating functional and morphological disorders that are strictly related to aspiration and can influence the decision about continued therapy [68].

Currently, there are no shared interventions to prevent swallowing and feeding problems; however, a specialized swallowing evaluation as well as consultation with speech therapist and nutritionist before starting full oral feeding is suggested to assess the safety of oral feeding and to reduce tracheal aspirations. Additionally, non-surgical management of dysphagia can be considered, although there is no focused therapy for this condition, and the treatment is dependent on the underlying cause [58]. In cases of severe malnutrition or aspiration during swallowing, temporary gastrostomy for aggressive nutritional management should be considered [39]. Generally, dysphagia has a good prognosis and improves with time. In a recent retrospective cohort study conducted in 111 patients with CEA, dysphagia was reported in 55% of patients <1 year, and in those aged between 5–11 years, the prevalence rate decreased to 17% and remained substantially stable in the age group 12–18 years. The severity of the disease tended to decrease with age [73].

## 8. Pulmonary Morbidity in Adulthood

Respiratory complications are very common in patients with repaired CEA ± TEF, although it is difficult to estimate the real prevalence of pulmonary involvement because of differing study designs and different surgical approaches over the years. In a prospective study conducted on 31 teenagers, Malmström et al. documented the occurrence of at least one pneumonic episode after surgical repair in 52% of cases [74]. Another study in 3479 patients with CEA ± TEF treated at 43 children’s hospitals documented 52% of hospital re-admissions for pneumonia in the first two years after the surgical repair, including 26% of patients with three or more pneumonia episodes [75]. A recent systematic first-year survey found occurrence of respiratory symptoms in 37% of the 307 CEA patients born in 2008 and 2009 and re-hospitalization for respiratory diseases in 28% [76]. Finally, a large observational study including 334 patients showed that the most hospital admissions due to respiratory illness occurred in children aged less than five years and that patients with GERD and low birth weight were more likely to be admitted [77].

Generally, although less frequently, recurrent respiratory infections tend to persist over time. However, wide differences in the prevalence of respiratory symptoms in adulthood has been reported, depending on the case series considered. Kovesi et al. reported a decrease over time in the prevalence of either bronchitis (75% in infancy and 40% over 15 years of age) or pneumonia (30% in infancy and 6% over 15 years) [28]. In a series of 101 adults treated for CEA between 1947 and 1985, 11% of patients reported current respiratory symptoms compared to 2% of healthy controls; a history of pneumonia and bronchitis were reported in 56% and 70% of patients (20% and 50% of controls), respectively [78]. Finally, in the study by Chetcuti et al., episodes of pneumonia after the age of 18 years were reported in 6% of the cases, and current bronchitis was reported in 14% of 125 adult patients born before 1969 [79].

The reasons for the persistence of respiratory morbidity to adulthood are not univocal (Table 3). The malformation itself, the acquired damage after the surgical repair, the various co-morbidities previously analyzed and the recurrence of lower respiratory tract infections at an early age can contribute to pulmonary impairment. Nevertheless, other conditions, including smoking habits and atopy can play a role in the recurrence of infections. Many studies have indicated an unusual high prevalence of atopy in patients with CEA ± Malmstrom et al. found that 17/31 (54%) of adolescents had a positive allergy skin prick test [74]. Sistonen et al. reported that 37/101 (37%) of CEA ± TEF cases had at least one positive reaction to common allergens in the skin prick test and that multiple sensitizations were associated with current respiratory symptoms [78]. Finally, more recently, Gatzinsky et al. found specific IgE in 11/28 (39%) adult patients who underwent CEA in 1968–1983 [80].

The mechanism by which patients with repaired CEA ± TEF have an increased prevalence of atopy or allergies is still controversial. However, the most reasonable hypothesis is a dysregulation of the respiratory epithelial barrier related to the malformation itself which can modify the tissue homeostasis and the innate immune response [81]. Additionally, bronchiectasis is a quite common finding. The onset of bronchiectasis is strictly related to recurrent pneumonia, and this condition has an incidence ranging from 14% to 17% in CEA ± TEF patients [82,83]. Bronchiectasis has been recognized as an irreversible condition which is, in turn, a risk factor for pulmonary exacerbations and respiratory impairment [84]. Additionally, chronic cough and/or barking cough are other very common conditions occurring in this population beginning in the first years of life and lasting to adulthood, but these symptoms seem to improve with age. In a study of 43 children born from 2011 to 2014, chronic cough was described in 72% of cases [85], whereas other studies of adolescents and adults reported a prevalence ranging from 19% to 34% [78,80,86].

Wheezing and dyspnea are frequent complaints of patients with repaired CEA ± TEF, and a higher incidence of doctor-diagnosed asthma has also been reported compared to the general population [78]. In the literature, the prevalence of asthma among patients with CEA varies between 16% and 30% [74,78,80]. However, a history of asthma-like symptoms, such as wheeze, recurrent cough, and breathlessness during respiratory infections, is not pathognomonic for bronchial asthma. Additionally, conditions such as tracheomalacia, airway malformations, or GERD may mimic asthma, which can be overestimated.

Many studies have described pulmonary function abnormalities in patients with repaired CEA ± TEF, including both restrictive and obstructive ventilatory patterns; other pediatric studies have instead reported normal lung function [80]. Interestingly, in a longitudinal study correlating respiratory symptoms, pulmonary function tests, and bronchial hyperreactivity with endobronchial biopsy performed at the age of <3, 3 to 7, and >7 years, the authors did not find signs of bronchial remodelling or chronic inflammation, which are typical changes that result from asthma. Despite recurrent respiratory infections, the presence of atopy and esophageal symptoms during childhood reduced pulmonary function during adolescence [74].

Our recent study of 13 children, operated on at a single center with a median 9-year follow-up (range 5–15 years) period, recorded a number of lower respiratory tract infections substantially similar to a control group of healthy children, although the infections were more severe in children with CEA ± TEF, who frequently needed hospitalization. An allergic sensitization was found in 23% of cases and in 7% of controls. The exhaled fraction of nitric oxide results were similar in the two groups (11 parts per billion, ppb, in cases and 12 ppb in controls), highlighting that the clinical relevance of nitric oxide was limited in this population. CEA patients had a mean forced vital capacity of 73% (104% in controls) and a mean forced expiratory volume in one second of 82% (95% in controls). Pulmonary function tests have only documented normal respiratory pattern or mild restrictive findings. No patient in the case group had an obstructive impairment. Residual volume and diffusion capacity were normal (96% and 103%, respectively), whereas the measurement of the resistance with the interruption technique has demonstrated higher baseline values in cases (230%) than in controls (104%), mostly without pharmacologic reversibility. It is likely that these findings are suggestive of narrowed airways and poor lung growth rather than of bronchial asthma, showing the importance of pulmonary function tests in these patients.

## 9. Conclusions

Currently, most children born with CEA ± TEF survive into adulthood, but many comorbidities, mainly the esophageal and respiratory issues, may persist throughout life. Pulmonary impairment involves many underlying mechanisms, which begin in the first years of life. This period coincides with a critical window for the growth of lungs and alveolar development. Therefore, early detection and management of pulmonary morbidity may be important to prevent impairment in pulmonary function and serious long-term complications. To obtain a successful outcome, it is fundamental to ensure a standardized, multidisciplinary follow-up that must continue until adulthood. A consensus protocol involving pediatricians, pulmonologists, allergists, gastroenterologists, otolaryngologists, surgeons, and physiotherapists is urgently needed to define the optimal multidisciplinary approach.

## Figures and Tables

**Table 1:** Reasons for the short-term pulmonary impairments and clinical manifestations in children with repaired congenital esophageal atresia and tracheoesophageal fistula.

Reasons	Clinical Manifestations
Anastomotic leaks	Serious systemic infections
	Severe pneumonia
	Cyanotic spells
	Atelectasis
	Pleural effusion
	Tension pneumothorax
Stricture	Dysphagia
	Regurgitation
	Aspiration pneumonia
	Chronic respiratory tract infections
Recurrence of fistula	Chronic chough
	Choking
	Cyanotic spells or cyanosis with feeding
	Dyspnoea
	Wheezing
	Recurrent pneumonia

**Table 2:** Long-term respiratory morbidity in patients with repaired congenital esophageal atresia and tracheoesophageal fistula.

Category	Clinical Presentation
Upper respiratory tract involvement	Tracheomalacia
	Vocal cord dysfunction
Lower respiratory tract involvement	Chest wall deformities
	Bronchiectasis
Gastrointestinal involvement	Esophagus dysmotility
	Gastro-esophageal reflux disease
Aspiration and dysphagia	Swallowing problems
	Feeding difficulties

**Table 3:** Reasons for the persistence of respiratory morbidity in patients with repaired congenital esophageal atresia and tracheoesophageal fistula.

Reasons
Malformation itself
Acquired damage after the surgical repair
Co-morbidities
Recurrence of lower respiratory tract infections
Atopy
Smoking habits
